# 4-[(*E*)-2-Ferrocenylethen­yl]-1,8-naphthalic anhydride

**DOI:** 10.1107/S1600536808007393

**Published:** 2008-03-29

**Authors:** Natasha H. Munro, Lyall R. Hanton, C. John McAdam, David A. McMorran

**Affiliations:** a Department of Chemistry, University of Otago, PO Box 56, Dunedin, New Zealand

## Abstract

In the structure of the title compound, [Fe(C_5_H_5_)(C_19_H_11_O_3_)], the plane of the substituted ferrocene ring is tilted by 14.17 (6)° with respect to the mean plane through the naphthalene ring system. In the crystal structure, centrosymmetric dimers are formed through π–π inter­actions [centroid–centroid distance = 3.624 (2) Å] between the substituted ferrocene ring and the three fused rings of the naphthalic anhydride unit. Pairs of dimers are held together by further naphthalene–naphthalene π–π interactions [distance between parallel mean planes 3.45 (3) Å]. Each dimer inter­acts with four neighbouring dimers in a herringbone fashion through C—H⋯π inter­actions, so forming a two-dimensional sheet-like structure.

## Related literature

For related literature, see: Allen (2002 ▶); Cuffe *et al.* (2005 ▶); Gan *et al.* (2004 ▶); Heck (1982 ▶); McAdam *et al.* (2003 ▶); Tian *et al.* (2000 ▶).
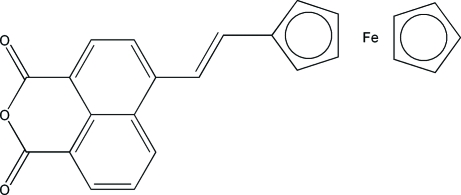


## Experimental

### 

#### Crystal data

[Fe(C_5_H_5_)(C_19_H_11_O_3_)]
*M* *_r_* = 408.22
Monoclinic, 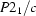

*a* = 10.1070 (6) Å
*b* = 10.0046 (6) Å
*c* = 16.8721 (10) Å
β = 92.878 (3)°
*V* = 1703.89 (18) Å^3^
*Z* = 4
Mo *K*α radiation
μ = 0.91 mm^−1^
*T* = 91 (2) K
0.38 × 0.14 × 0.09 mm

#### Data collection

Bruker APEXII CCD area-detector diffractometer
Absorption correction: multi-scan (*SADABS*; Bruker, 2006 ▶) *T*_min_ = 0.816, *T*_max_ = 0.921
26830 measured reflections
2984 independent reflections
2895 reflections with *I* > 2σ(*I*)
*R*_int_ = 0.027

#### Refinement

*R*[*F* ^2^ > 2σ(*F* ^2^)] = 0.036
*wR*(*F* ^2^) = 0.089
*S* = 1.10
2984 reflections
253 parameters
H-atom parameters constrained
Δρ_max_ = 0.90 e Å^−3^
Δρ_min_ = −0.26 e Å^−3^

### 

Data collection: *APEX2* (Bruker, 2006 ▶); cell refinement: *APEX2*; data reduction: *SAINT* (Bruker, 2006 ▶); program(s) used to solve structure: *SIR92* (Altomare *et al.*, 1993 ▶); program(s) used to refine structure: *SHELXL97* (Sheldrick, 2008 ▶); molecular graphics: *SHELXTL* (Sheldrick, 2008 ▶) and *Mercury* (Macrae *et al.*, 2006 ▶); software used to prepare material for publication: *SHELXL97* and *enCIFer* (Allen *et al.*, 2004 ▶).

## Supplementary Material

Crystal structure: contains datablocks global, I. DOI: 10.1107/S1600536808007393/su2047sup1.cif

Structure factors: contains datablocks I. DOI: 10.1107/S1600536808007393/su2047Isup2.hkl

Additional supplementary materials:  crystallographic information; 3D view; checkCIF report

## Figures and Tables

**Table 1:** C—H⋯π geometry (Å, °)

*D*—H⋯*A*	*D*—H	H⋯*A*	*D*⋯*A*	*D*—H⋯*A*
C18—H18⋯*Cg*^i^	0.93	2.77	3.449 (3)	131

Symmetry code: (i) 
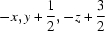
. *Cg* is the centroid of atoms C20–C24.

**Fig. 1.**
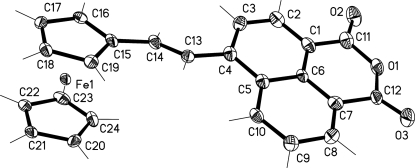
The molecular structure of compound I, showing the atom numbering scheme and displacement ellipsoids drawn at the 50% probability level.

**Fig. 2.**
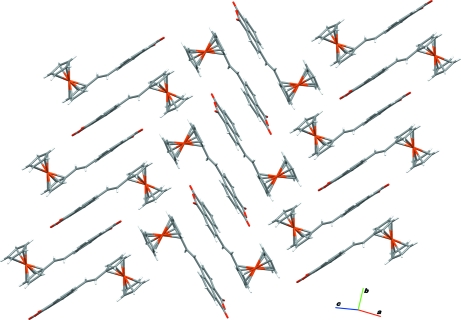
Crystal packing in compound I, showing the formation of the two-dimensional sheet-like structure with the herring-bone packing.

## supplementary crystallographic information

### Comment

Molecular dyads have potential uses in the development of new molecular materials for a variety of technological applications (Gan *et al.*, 2004; Tian *et al.*, 2000). A group of attractive donor-acceptor targets involve species incorporating oxidisable ferrocene moieties and fluorescent naphthalamide acceptors (Cuffe *et al.*, 2005).

The title compound, I, was prepared by the Heck coupling (Heck 1982) of 4-bromonaphthalic anhydride and vinylferrocene. Cyclic voltametry of I shows a reversible ferrocenyl oxidation at 0.62 V and an irreversible multi-electron process at -1.0 V associated with the naphthalene moiety (McAdam *et al.*, 2003).

The molecular structure of compound I is illustrated in Fig. 1. The molecule consists of a ferrocene moiety linked in a *trans* fashion through an ethene to a naphthalic anhydride group. The bond length between the ethene carbons, C13 and C14, agrees well with the mean value of 1.32 (2) Å found for 49 observations in 41 structures from the Cambridge Structural Database (version 5.28, last update Nov. 2007; Allen, 2002). The ferrocene rings are eclipsed and tilted by 3.14 (9)° with respect to each other. The plane of the substituted ferrocene ring is tilted by 14.17 (6)° with respect to the plane of the naphthalene ring [C1—C12, O1] such that the ethene linked ring is above the plane. This tilt arises from steric interactions between the *peri* H atom of the naphthalene ring and the adjacent H atom of the ethene moiety.

In the crystal structure of I centrosymmetric dimers are formed through strong complementary π-π interactions between the substituted ferrocene rings and the heterocyclic pyrandione rings [C1, C6, C7, C11, C12, O1]. The distance between the centroids of the cyclopentadiene and the six-membered heterocyclic ring is 3.624 (2) Å. These dimers are held together by naphthalene-naphthalene π-π interactions, with a distance of 3.45 (3) Å between the symmetry related parallel mean planes of the three fused rings of the naphthalic anhydride moiety. In addition there are also complementary *C*=*O*···*H*—*C*(cyclopentadiene) interactions at 2.46 Å [corresponding separation for O3···C24 is 3.386 (4) Å]. Packing is achieved through (cyclopentadiene) C—H···π(cyclopentadiene) interactions at 2.77 Å, such that each dimer interacts with four neighbouring dimers in a herringbone fashion to form a two-dimensional sheet-like structure (Fig. 2).

### Experimental

The reagents 4-bromonaphthalic anhydride (137.6 mg, 0.497 mmol), vinylferrocene (95.4 mg, 0.45 mmol), Pd(OAc)_2_ (63.9 mg, 0.285 mmol) and *P*(*o*-tolyl)_3_ (21.5 mg, 0.071 mmol) were combined then dissolved in DMF (20 ml) with Et_3_N (0.3 ml, 2.16 mmol). The dark red solution obtained was refluxed for 18 h at 80°C giving a dark brown solution. This was reduced in volume to give a black oil and solid. The crude mixture was purified by column chromatography (DCM on 5% hydrated SiO_2_) and the band which eluted fourth was reduced to give 28.4 mg (16%) of a green/black solid, compound I. NMR (δ, p.p.m., CDCl_3_): 4.22 (s, 5H, Cp) 4.47 (t, 2H (J = 2 Hz) Hβ) 4.63 (t, 2H, (J = 2 Hz) Hα) 7.28 (d, 1H (J = 16) Fc—CH=CH—*R*) 7.47 (d, 1H (J = 16 Hz) Fc—CH=CH—*R*) 7.84 (dd, 1H (J = 7, 9 Hz) H-6) 8.01 (d, 1H (J = 8 Hz) H-3) 8.59 (d, 1H (J = 8 Hz) H-2) 8.64 (m, 2H, H-5, H-7). IR (KBr, cm^-1^): 1768, 1725 (ν_c=o_).E (CH_2_Cl_2_, V): [Fc]^+/0^ = 0.62, [naph]^0/-^ = -1.0 (irreversible multi-electron process). UV-vis (λ, nm (ε, mol^-1^*L* cm^-1^*x* 10^-3^), CH_2_Cl_2_): compound (I) 549 (6), 394 (16), 298 (8); oxidized compound (I) 834 (0.9), 386 (19). High resolution mass spec {*M*^+^] predicted: 408.04299. Found: 408.04299. Anal. Calc. for C_24_H_16_FeO_3_: C, 70.61; H, 3.95. Found: C, 70.70; H, 3.96. X-ray quality crystals were grown by the slow evaporation of an acetonitrile solution.

### Refinement

The H-atoms were included in calculated positions and treated as riding atoms: d(C—H) = 0.93 Å with *U*_iso_=1.2*U*_eq_(C).

### Crystal data

**Table:**

[Fe(C_5_H_5_)(C_19_H_11_O_3_)]	*F*_000_ = 840
*M**_r_* = 408.22	*D*_x_ = 1.591 Mg m^−^^3^
Monoclinic, *P*2_1_/*c*	Mo *K*α radiation λ = 0.71073 Å
Hall symbol: -P 2ybc	Cell parameters from 9917 reflections
*a* = 10.1070 (6) Å	θ = 2.9–37.6º
*b* = 10.0046 (6) Å	µ = 0.91 mm^−^^1^
*c* = 16.8721 (10) Å	*T* = 91 (2) K
β = 92.878 (3)º	Rhomb, black
*V* = 1703.89 (18) Å^3^	0.38 × 0.14 × 0.09 mm
*Z* = 4	

### Data collection

**Table:**

Bruker APEXII CCD area-detector diffractometer	2984 independent reflections
Radiation source: fine-focus sealed tube	2895 reflections with *I* > 2σ(*I*)
Monochromator: graphite	*R*_int_ = 0.027
*T* = 91(2) K	θ_max_ = 25.0º
φ and ω scans	θ_min_ = 2.4º
Absorption correction: multi-scan(SADABS; Bruker, 2006)	*h* = −12→12
*T*_min_ = 0.816, *T*_max_ = 0.921	*k* = −11→11
26830 measured reflections	*l* = −20→19

### Refinement

**Table:**

Refinement on *F*^2^	Secondary atom site location: difference Fourier map
Least-squares matrix: full	Hydrogen site location: inferred from neighbouring sites
*R*[*F*^2^ > 2σ(*F*^2^)] = 0.036	H-atom parameters constrained
*wR*(*F*^2^) = 0.089	*w* = 1/[σ^2^(*F*_o_^2^) + (0.0331*P*)^2^ + 3.1715*P*] where *P* = (*F*_o_^2^ + 2*F*_c_^2^)/3
*S* = 1.10	(Δ/σ)_max_ = 0.002
2984 reflections	Δρ_max_ = 0.90 e Å^−^^3^
253 parameters	Δρ_min_ = −0.26 e Å^−^^3^
Primary atom site location: structure-invariant direct methods	Extinction correction: none

### Special details

**Table:**

Experimental. Spectroelectrochemical measurements were obtained with a Pt electrode in CH_2_Cl_2_ with 0.1 *M* TBAPF_6_ at 20°C, referenced against decamethylferrocene where [Fc]^+/0^ = 0.55 V. UV-vis spectra were performed in an UV-vis OTTLE cell (3 mm volume) with a Pt grid and auxilary electrodes in CHCl2 at 20°C, referenced against internal Ag wire.
Geometry. All e.s.d.'s (except the e.s.d. in the dihedral angle between two l.s. planes) are estimated using the full covariance matrix. The cell e.s.d.'s are taken into account individually in the estimation of e.s.d.'s in distances, angles and torsion angles; correlations between e.s.d.'s in cell parameters are only used when they are defined by crystal symmetry. An approximate (isotropic) treatment of cell e.s.d.'s is used for estimating e.s.d.'s involving l.s. planes.
Refinement. Refinement of *F*^2^ against ALL reflections. The weighted *R*-factor *wR* and goodness of fit *S* are based on *F*^2^, conventional *R*-factors *R* are based on *F*, with *F* set to zero for negative *F*^2^. The threshold expression of *F*^2^ > σ(*F*^2^) is used only for calculating *R*-factors(gt) *etc*. and is not relevant to the choice of reflections for refinement. *R*-factors based on *F*^2^ are statistically about twice as large as those based on *F*, and *R*- factors based on ALL data will be even larger.

### Fractional atomic coordinates and isotropic or equivalent isotropic displacement parameters (Å^2^)

**Table:**

	*x*	*y*	*z*	*U*_iso_*/*U*_eq_	
Fe1	0.07515 (3)	1.05807 (3)	0.660712 (19)	0.01502 (12)	
O1	0.69220 (18)	0.43886 (18)	0.35524 (11)	0.0251 (4)	
C6	0.5911 (2)	0.6272 (2)	0.45911 (14)	0.0169 (5)	
C4	0.4346 (2)	0.8070 (2)	0.48669 (15)	0.0204 (5)	
C5	0.5405 (2)	0.7223 (2)	0.51375 (15)	0.0199 (5)	
C13	0.3814 (2)	0.9090 (2)	0.53817 (15)	0.0190 (5)	
H13	0.4355	0.9392	0.5807	0.023*	
C15	0.2091 (2)	1.0720 (2)	0.57412 (15)	0.0200 (5)	
C19	0.2614 (2)	1.1284 (2)	0.64766 (15)	0.0190 (5)	
H19	0.3372	1.1005	0.6766	0.023*	
O3	0.8461 (2)	0.3763 (2)	0.44491 (12)	0.0348 (5)	
C22	−0.0984 (2)	1.0294 (3)	0.71692 (15)	0.0204 (5)	
H22	−0.1681	1.0897	0.7191	0.025*	
C1	0.5384 (2)	0.6172 (3)	0.38121 (15)	0.0223 (5)	
C23	−0.0840 (3)	0.9306 (3)	0.65806 (16)	0.0251 (6)	
H23	−0.1426	0.9145	0.6149	0.030*	
C10	0.5973 (2)	0.7270 (2)	0.59164 (14)	0.0201 (5)	
H10	0.5643	0.7867	0.6280	0.024*	
C12	0.7539 (2)	0.4460 (3)	0.42967 (16)	0.0223 (5)	
C16	0.0908 (2)	1.1446 (3)	0.55162 (15)	0.0210 (5)	
H16	0.0358	1.1288	0.5068	0.025*	
C17	0.0725 (2)	1.2437 (2)	0.60898 (15)	0.0217 (5)	
H17	0.0034	1.3052	0.6082	0.026*	
C20	0.0949 (3)	0.9163 (2)	0.74713 (15)	0.0221 (5)	
H20	0.1741	0.8893	0.7727	0.026*	
O2	0.5549 (2)	0.5067 (2)	0.25773 (12)	0.0355 (5)	
C8	0.7537 (3)	0.5533 (3)	0.56121 (16)	0.0262 (6)	
H8	0.8251	0.4992	0.5769	0.031*	
C14	0.2605 (2)	0.9620 (2)	0.52849 (15)	0.0206 (5)	
H14	0.2041	0.9254	0.4890	0.025*	
C11	0.5911 (3)	0.5219 (3)	0.32662 (17)	0.0265 (6)	
C21	0.0111 (2)	1.0207 (2)	0.77156 (14)	0.0186 (5)	
H21	0.0260	1.0743	0.8161	0.022*	
C2	0.4360 (2)	0.7034 (3)	0.35592 (16)	0.0254 (6)	
H2	0.4008	0.6987	0.3040	0.030*	
C7	0.6983 (2)	0.5456 (2)	0.48600 (15)	0.0214 (5)	
C3	0.3872 (2)	0.7951 (3)	0.40754 (15)	0.0232 (5)	
H3	0.3196	0.8519	0.3891	0.028*	
C18	0.1760 (2)	1.2345 (2)	0.66792 (15)	0.0200 (5)	
H18	0.1865	1.2888	0.7125	0.024*	
C24	0.0358 (3)	0.8603 (2)	0.67643 (16)	0.0275 (6)	
H24	0.0696	0.7902	0.6474	0.033*	
C9	0.7014 (3)	0.6443 (3)	0.61456 (16)	0.0265 (6)	
H9	0.7375	0.6489	0.6663	0.032*	

### Atomic displacement parameters (Å^2^)

**Table:**

	*U*^11^	*U*^22^	*U*^33^	*U*^12^	*U*^13^	*U*^23^
Fe1	0.0178 (2)	0.01168 (19)	0.0162 (2)	−0.00187 (13)	0.00728 (13)	0.00029 (13)
O1	0.0288 (10)	0.0219 (9)	0.0255 (10)	0.0002 (8)	0.0084 (8)	−0.0089 (7)
C6	0.0169 (11)	0.0120 (11)	0.0227 (12)	−0.0051 (9)	0.0096 (10)	0.0003 (9)
C4	0.0183 (12)	0.0173 (12)	0.0261 (13)	−0.0048 (10)	0.0065 (10)	−0.0015 (10)
C5	0.0197 (12)	0.0160 (12)	0.0245 (13)	−0.0054 (10)	0.0072 (10)	−0.0009 (10)
C13	0.0194 (12)	0.0171 (12)	0.0210 (12)	−0.0012 (10)	0.0052 (10)	0.0023 (10)
C15	0.0252 (13)	0.0141 (12)	0.0221 (13)	−0.0059 (10)	0.0143 (10)	0.0005 (10)
C19	0.0157 (11)	0.0179 (12)	0.0241 (13)	−0.0032 (9)	0.0063 (10)	0.0040 (10)
O3	0.0388 (12)	0.0286 (11)	0.0378 (12)	0.0101 (9)	0.0102 (9)	−0.0033 (9)
C22	0.0191 (12)	0.0217 (13)	0.0212 (13)	−0.0014 (10)	0.0094 (10)	0.0058 (10)
C1	0.0198 (12)	0.0243 (13)	0.0232 (13)	−0.0084 (10)	0.0063 (10)	−0.0033 (10)
C23	0.0271 (13)	0.0281 (14)	0.0205 (13)	−0.0138 (11)	0.0062 (11)	0.0024 (11)
C10	0.0247 (13)	0.0179 (12)	0.0182 (12)	−0.0039 (10)	0.0066 (10)	−0.0039 (10)
C12	0.0195 (12)	0.0217 (13)	0.0266 (14)	0.0012 (11)	0.0095 (10)	0.0022 (10)
C16	0.0222 (12)	0.0221 (13)	0.0193 (12)	−0.0024 (10)	0.0078 (10)	0.0048 (10)
C17	0.0232 (13)	0.0164 (12)	0.0266 (14)	0.0026 (10)	0.0118 (11)	0.0064 (10)
C20	0.0215 (12)	0.0180 (12)	0.0274 (14)	0.0026 (10)	0.0086 (10)	0.0089 (10)
O2	0.0325 (11)	0.0474 (13)	0.0267 (11)	−0.0013 (9)	0.0030 (9)	−0.0164 (9)
C8	0.0267 (13)	0.0236 (13)	0.0285 (14)	0.0048 (11)	0.0017 (11)	0.0029 (11)
C14	0.0225 (12)	0.0188 (12)	0.0211 (13)	−0.0038 (10)	0.0086 (10)	−0.0015 (10)
C11	0.0205 (13)	0.0267 (14)	0.0327 (16)	−0.0074 (11)	0.0059 (11)	−0.0045 (12)
C21	0.0234 (12)	0.0162 (12)	0.0169 (12)	−0.0024 (10)	0.0080 (10)	0.0023 (9)
C2	0.0198 (12)	0.0335 (15)	0.0227 (13)	−0.0056 (11)	0.0006 (10)	−0.0035 (11)
C7	0.0233 (13)	0.0179 (12)	0.0234 (13)	−0.0063 (10)	0.0039 (10)	0.0010 (10)
C3	0.0183 (12)	0.0257 (13)	0.0255 (14)	0.0017 (10)	−0.0014 (10)	0.0003 (11)
C18	0.0270 (13)	0.0131 (11)	0.0207 (13)	−0.0058 (10)	0.0114 (10)	−0.0021 (9)
C24	0.0422 (16)	0.0110 (12)	0.0316 (14)	−0.0049 (11)	0.0237 (12)	−0.0008 (10)
C9	0.0316 (14)	0.0237 (13)	0.0245 (14)	0.0039 (11)	0.0041 (11)	−0.0005 (11)

### Geometric parameters (Å, °)

**Table:**

Fe1—C19	2.032 (2)	C22—C23	1.414 (4)
Fe1—C20	2.037 (2)	C22—H22	0.9300
Fe1—C24	2.038 (2)	C1—C2	1.398 (4)
Fe1—C18	2.039 (2)	C1—C11	1.446 (4)
Fe1—C21	2.044 (2)	C23—C24	1.421 (4)
Fe1—C15	2.046 (2)	C23—H23	0.9300
Fe1—C16	2.047 (2)	C10—C9	1.378 (4)
Fe1—C23	2.051 (3)	C10—H10	0.9300
Fe1—C17	2.052 (2)	C12—C7	1.505 (4)
Fe1—C22	2.055 (2)	C16—C17	1.404 (4)
O1—C12	1.376 (3)	C16—H16	0.9300
O1—C11	1.385 (3)	C17—C18	1.410 (4)
C6—C1	1.397 (4)	C17—H17	0.9300
C6—C7	1.413 (4)	C20—C21	1.419 (3)
C6—C5	1.436 (3)	C20—C24	1.422 (4)
C4—C3	1.401 (4)	C20—H20	0.9300
C4—C5	1.423 (4)	O2—C11	1.210 (3)
C4—C13	1.460 (3)	C8—C7	1.363 (4)
C5—C10	1.408 (4)	C8—C9	1.402 (4)
C13—C14	1.334 (4)	C8—H8	0.9300
C13—H13	0.9300	C14—H14	0.9300
C15—C16	1.433 (4)	C21—H21	0.9300
C15—C19	1.439 (4)	C2—C3	1.374 (4)
C15—C14	1.454 (3)	C2—H2	0.9300
C19—C18	1.421 (3)	C3—H3	0.9300
C19—H19	0.9300	C18—H18	0.9300
O3—C12	1.182 (3)	C24—H24	0.9300
C22—C21	1.407 (4)	C9—H9	0.9300
			
C19—Fe1—C20	105.12 (10)	C23—C22—H22	125.9
C19—Fe1—C24	122.55 (11)	Fe1—C22—H22	126.4
C20—Fe1—C24	40.84 (11)	C6—C1—C2	119.0 (2)
C19—Fe1—C18	40.87 (10)	C6—C1—C11	120.8 (2)
C20—Fe1—C18	121.99 (11)	C2—C1—C11	120.2 (2)
C24—Fe1—C18	158.90 (12)	C22—C23—C24	108.0 (2)
C19—Fe1—C21	120.01 (10)	C22—C23—Fe1	70.01 (14)
C20—Fe1—C21	40.69 (10)	C24—C23—Fe1	69.17 (14)
C24—Fe1—C21	68.32 (10)	C22—C23—H23	126.0
C18—Fe1—C21	106.50 (10)	C24—C23—H23	126.0
C19—Fe1—C15	41.34 (10)	Fe1—C23—H23	126.4
C20—Fe1—C15	120.94 (10)	C9—C10—C5	120.7 (2)
C24—Fe1—C15	107.42 (10)	C9—C10—H10	119.6
C18—Fe1—C15	68.72 (9)	C5—C10—H10	119.6
C21—Fe1—C15	156.53 (11)	O3—C12—O1	119.0 (2)
C19—Fe1—C16	69.03 (10)	O3—C12—C7	124.6 (3)
C20—Fe1—C16	158.25 (10)	O1—C12—C7	116.4 (2)
C24—Fe1—C16	123.50 (11)	C17—C16—C15	108.2 (2)
C18—Fe1—C16	68.07 (10)	C17—C16—Fe1	70.12 (14)
C21—Fe1—C16	160.44 (10)	C15—C16—Fe1	69.43 (14)
C15—Fe1—C16	40.99 (10)	C17—C16—H16	125.9
C19—Fe1—C23	160.48 (11)	C15—C16—H16	125.9
C20—Fe1—C23	68.35 (11)	Fe1—C16—H16	126.1
C24—Fe1—C23	40.66 (11)	C16—C17—C18	108.7 (2)
C18—Fe1—C23	158.20 (11)	C16—C17—Fe1	69.80 (14)
C21—Fe1—C23	67.81 (10)	C18—C17—Fe1	69.36 (14)
C15—Fe1—C23	124.95 (10)	C16—C17—H17	125.6
C16—Fe1—C23	109.84 (10)	C18—C17—H17	125.6
C19—Fe1—C17	68.47 (10)	Fe1—C17—H17	126.8
C20—Fe1—C17	159.02 (11)	C21—C20—C24	107.6 (2)
C24—Fe1—C17	159.33 (12)	C21—C20—Fe1	69.93 (14)
C18—Fe1—C17	40.31 (10)	C24—C20—Fe1	69.64 (14)
C21—Fe1—C17	123.83 (10)	C21—C20—H20	126.2
C15—Fe1—C17	68.26 (10)	C24—C20—H20	126.2
C16—Fe1—C17	40.07 (10)	Fe1—C20—H20	125.8
C23—Fe1—C17	124.05 (11)	C7—C8—C9	118.9 (2)
C19—Fe1—C22	156.20 (10)	C7—C8—H8	120.5
C20—Fe1—C22	68.13 (10)	C9—C8—H8	120.5
C24—Fe1—C22	68.15 (10)	C13—C14—C15	125.8 (2)
C18—Fe1—C22	121.92 (10)	C13—C14—H14	117.1
C21—Fe1—C22	40.14 (10)	C15—C14—H14	117.1
C15—Fe1—C22	161.72 (11)	O2—C11—O1	116.2 (2)
C16—Fe1—C22	125.47 (10)	O2—C11—C1	126.2 (3)
C23—Fe1—C22	40.27 (10)	O1—C11—C1	117.5 (2)
C17—Fe1—C22	109.22 (10)	C22—C21—C20	108.4 (2)
C12—O1—C11	125.4 (2)	C22—C21—Fe1	70.36 (14)
C1—C6—C7	120.7 (2)	C20—C21—Fe1	69.38 (14)
C1—C6—C5	121.4 (2)	C22—C21—H21	125.8
C7—C6—C5	117.9 (2)	C20—C21—H21	125.8
C3—C4—C5	118.1 (2)	Fe1—C21—H21	126.1
C3—C4—C13	120.5 (2)	C3—C2—C1	120.2 (2)
C5—C4—C13	121.3 (2)	C3—C2—H2	119.9
C10—C5—C4	123.0 (2)	C1—C2—H2	119.9
C10—C5—C6	118.7 (2)	C8—C7—C6	122.6 (2)
C4—C5—C6	118.3 (2)	C8—C7—C12	118.5 (2)
C14—C13—C4	124.5 (2)	C6—C7—C12	119.0 (2)
C14—C13—H13	117.7	C2—C3—C4	123.0 (2)
C4—C13—H13	117.7	C2—C3—H3	118.5
C16—C15—C19	107.2 (2)	C4—C3—H3	118.5
C16—C15—C14	124.0 (2)	C17—C18—C19	108.5 (2)
C19—C15—C14	128.9 (2)	C17—C18—Fe1	70.33 (14)
C16—C15—Fe1	69.57 (13)	C19—C18—Fe1	69.30 (13)
C19—C15—Fe1	68.82 (13)	C17—C18—H18	125.7
C14—C15—Fe1	126.43 (17)	C19—C18—H18	125.7
C18—C19—C15	107.4 (2)	Fe1—C18—H18	126.2
C18—C19—Fe1	69.83 (13)	C23—C24—C20	107.8 (2)
C15—C19—Fe1	69.84 (13)	C23—C24—Fe1	70.16 (14)
C18—C19—H19	126.3	C20—C24—Fe1	69.53 (14)
C15—C19—H19	126.3	C23—C24—H24	126.1
Fe1—C19—H19	125.6	C20—C24—H24	126.1
C21—C22—C23	108.2 (2)	Fe1—C24—H24	125.8
C21—C22—Fe1	69.50 (14)	C10—C9—C8	121.1 (3)
C23—C22—Fe1	69.72 (14)	C10—C9—H9	119.4
C21—C22—H22	125.9	C8—C9—H9	119.4

### Hydrogen-bond geometry (Å, °)

**Table:**

*D*—H···*A*	*D*—H	H···*A*	*D*···*A*	*D*—H···*A*
C18—H18···Cg^i^	0.93	2.77	3.449 (3)	131

Symmetry codes: (i) −*x*, *y*+1/2, −*z*+3/2.
